# Isoorientin Attenuates Cisplatin-Induced Nephrotoxicity Through the Inhibition of Oxidative Stress and Apoptosis via Activating the SIRT1/SIRT6/Nrf-2 Pathway

**DOI:** 10.3389/fphar.2020.00264

**Published:** 2020-03-18

**Authors:** Xiaoye Fan, Wei Wei, Jingbo Huang, Xingkai Liu, Xinxin Ci

**Affiliations:** ^1^Institute of Translational Medicine, The First Hospital of Jilin University, Changchun, China; ^2^Department of Urology, The First Hospital of Jilin University, Changchun, China; ^3^Department of Traditional Chinese Medicine, The First Hospital of Jilin University, Changchun, China; ^4^Department of Hepatopancreatobiliary Surgery, The First Hospital of Jilin University, Changchun, China

**Keywords:** Isoorientin, cisplatin, oxidative stress, nephrotoxicity, Nrf2

## Abstract

Cisplatin (CDDP) is a widely used chemotherapeutic agent for various solid tumors, but its severe side effects, particularly nephrotoxicity, limit its clinical application. Isoorientin (Iso) is a flavonoid-like compound known to have antioxidant effects. As oxidative injury plays a vital role in CDDP-induced acute kidney injury (AKI), the effect of Iso on CDDP-induced nephrotoxicity has not yet been researched. We assessed the effects of Iso against CDDP-induced nephrotoxicity *in vitro* using mTEC cells and further explored the mechanisms underlying CDDP-induced renal dysfunction *in vivo* in WT and Nrf2^–/–^ mice. The results showed that Iso treatment significantly reduced CDDP-induced nephrotoxicity via attenuating cell damage *in vitro* and via ameliorating renal injury, as determined by biochemical markers, in mice. The molecular mechanism underlying this protection was also investigated. Iso up-regulated the expression levels of SIRT1 and SIRT6 *in vivo* and *in vitro*. In addition, Iso activated Nrf2 translocation and the expression levels of its downstream antioxidant enzymes, such as HO-1 and NQO1, whereas it inhibited the expression level of NOX4, thus decreasing oxidative stress. Notably, the protective effects of Iso observed in WT mice were completely abolished in Nrf2^–/–^ mice. Collectively, these data indicate that the protective effect of Iso on CDDP-induced nephrotoxicity by SIRT1- and SIRT6-mediated Nrf2 activation regulates oxidative stress, inflammation and apoptosis. The absence of Nrf2 exacerbates CDDP-induced renal damage, and the pharmacological activation of Nrf2 may represent a novel therapy to prevent kidney injury.

## Introduction

Although cisplatin (CDDP) is an effective chemotherapeutic drug for the treatment of different types of human tumors, its clinical application is limited owing to its serious side effects, especially nephrotoxicity. Although saline hydration and diuresis during CDDP administration reduces the rate of these effects to approximately a quarter of the baseline rate (Hartmann et al., 1999) strategies to prevent or treat CDDP-induced AKIare lacking. Thus, considerable motivation exists to identify the molecular targets implicated in CDDP-induced AKI.

The mechanism of CDDP-induced side effects involves oxidative stress, DNA adduct formation, mitochondrial dysfunction, caspase activation, apoptotic and/or necrotic cell death, inflammatory responses (Pan et al., 2009; Nozaki et al., 2011; Oh et al., 2014; Sahu et al., 2014). Among several mechanisms of CDDP-induced renal damage, oxidative stress-mediated nephrotoxicity is significant. The overproduction of ROS can induce inflammation, mitochondrial dysfunction and cell apoptosis (Reuter et al., 2010; Wu et al., 2018). Therefore, counteracting the environmental stress caused by these reactive species can further inhibit inflammation and apoptosis and thus play a protective role in CDDP-induced renal injury. Multiple research groups have also proved that mitogen-activated protein kinase (MAPK) pathways are involved in the development and progression of CDDP-induced kidney injury (Guerrero-Beltran et al., 2012). Many CDDP-induced nephrotoxic animal studies have also emphasized the strong contribution of inflammation to pathogenesis (Pan et al., 2009; Rajasundari et al., 2011). In addition, ROS are important for enhancing inflammation response by activating NF-κB and its related signaling pathways (Sahin et al., 2010; Oh et al., 2014). Additionally, a substantial body of literature has documented the role of Nrf2 in the regulation of physiological processes, which inhibits the development and progression of CDDP-induced renal injury (Park et al., 2008; Shelton et al., 2013). Deficiency of Nrf2 reportedly exacerbates CDDP-mediated nephrotoxicity and it has been shown that the pharmacological activation of Nrf2 inhibits CDDP-mediated nephrotoxicity (Aleksunes et al., 2010; Shelton et al., 2013). Therefore, important molecular target to prevent nephrotoxicity caused by CDDP is considered to be the pharmacological activation of Nrf2.

SIRT1 is one of the seven mammalian homologs of Sir2 (Sirt1–7). It is located in the nucleus and regulates nucleosome histone acetylation and the activity of several transcriptional factors to exert cytoprotective effects through the inhibition of apoptosis, inflammation, and fibrosis (Vaquero et al., 2004). SIRT1 is widely expressed in kidney, such as tubular cells and podocytes. Previous studies have shown that SIRT1 deficits in the kidneys of aged mice result in an increased susceptibility to CDDP-induced AKI (Guan et al., 2017). SIRT6 is also located in the nucleus, responds to oxidative stress and has been shown to be recruited to sites of DNA double-strand breaks and promote DNA repair through ADP-ribosylation (Lerrer et al., 2016). Additionally, SIRT6 overexpression can inhibit ERK1/2 expression and alleviate CDDP-induced AKI, thereby suppressing NF-κB and p53 signaling (Li et al., 2018). Recent research has shown that SIRT1 may function by regulating oxidative stress and antioxidant enzymes, while SIRT6 can protect cells from oxidative stress-associated DNA damage, thus increasing the possibility that SIRT1 and SIRT6 are active modulators of the Nrf2 antioxidant pathway (Mao et al., 2011; Meng et al., 2015).

Isoorientin (3′,4′,5,7-tetrahydroxy-6-C-glucopyranosyl flav- one; Iso) is a C-glycosyl flavone commonly found in human diet. It has been extracted from several plant species, including *Phyllostachys pubescens*, *Gentiana*, *Patrinia*, buckwheat (Yuan et al., 2012). Previous studies have been shown that Iso exerts various pharmacological activities, such as anti-inflammatory activities and antioxidant activities (Anilkumar et al., 2017). And, we found that Iso ameliorates APAP-induced hepatotoxicity via the activation of the Nrf2 antioxidative pathway and the involvement of AMPK/Akt/GSK3β (Fan et al., 2018). Natural products are the main Nrf2 activators, which can regulate the Nrf2/ARE pathway to eliminate oxidative stress, and have attracted increasing attention in recent years. However, the protective effect of Iso against CDDP-induced nephrotoxicity and the underlying molecular mechanisms have not previously been investigated. Therefore, the aim of this study was to explore the protective effects of Iso in CDDP-induced nephrotoxicity and the molecular mechanisms and the potential targets involved.

## Materials and Methods

### Reagents and Chemical

Iso, purity >98%) was supplied by Chengdu Pufei De Biotech CO., Ltd., Hoechst 33342 was offered by Invitrogen (Carlsbad, CA, United States). Antibodies against Nrf2, NOX4, HO-1, NQO1, and SIRT Activity Assay Kit were purchased from Abcam (Cambridge, MA, United States). Antibody against SIRT1 was purchased from Santa Cruz (CA, United States). Antibody against SIRT6 was purchased from Elabscience (Wuhan, China). CDDP and the inhibitors of SIRT1 and SIRT6 were purchased from Medchem express (NJ, United States). Antibodies against p65, p-JNK, p-ERK, p-p38, HMGB1, Ac-p53, p53, p-p53, BAX, BCL-2, Caspase-3, β-actin, GAPDH, Lamin B were purchased from Cell Signaling (Boston, MA, United States). The horseradish peroxidase (HRP)-conjugated anti-rabbit and anti-mouse IgG were purchased from proteintech (Boston, MA, United States). Prime-Script RT-PCR kit and Faststart Universal SYBR Green Master were offered by Takara (Dalian, China) and Roche (Basel, Switzerland), respectively. Tetraethylbenzimidazolylcarbocyanine iodide (JC-1) was obtained from the Beyotime Institute of Biotechnology (Shanghai, China). Additionally, BUN, CRE, GSH, MPO, MDA and SOD test kits were offered by Nanjing Jiancheng Bioengineering Institute (Nanjing, China).

### Cell Culture and Cell Viability Assay

Mouse renal tubular epithelial cells (mTECs) purchased from the China Cell Line Bank (Beijing, China) and cultured at 37°C in a 5% CO_2_ atmosphere in DMEM supplemented with 10% FBS and antibiotics(100 U/ml of penicillin, 100 μg/ml of streptomycin). For cell viability, mTECs cells grown in 96-well plates (1.5 × 10^4^ cells/well) were incubated with Iso (5, 10, or 20 μM) for 1 h, and treated with CDDP (20 μM) for 24 h. Then, cell viability was measured using Cell Counting Kit-8 assay and normalized as the percentage of control.

### Hoechst 33342/PI Staining

mTECs grown in 12-well plates. After treatments, cells were incubated with 5 μg/ml Hoechst 33342 (Invitrogen, United States) and 15 μg/ml PI (Sigma, St Louis, United States) for 15 min at 37°C and then washed with PBS. The fluorescence was immediately detected using a fluorescence microscope.

### Real-Time Polymerase Chain Reaction (qPCR)

Total RNA was extracted from cultured cells with Trizol reagent, and reversely transcripted to cDNA using the Prime-Script RT-PCR kit (Takara) according to the manufacturer’s procedure. Relative change in mRNA level was calculated by the comparative Ct method, using β-actin served as control.

### Quantification of SIRT1 Activity

SIRT1 activity from mTECs were quantified using commercially available kits according to the manufacturer’s procedure (Abcam).

### Measurement of ROS Production

The intracellular ROS level was measured by a fluorometric assay. mTECs grown in 96-well plates (1.5 × 10^4^ cells/well) were performed to Iso (5, 10 or 20 μM) for 18 h, subsequently treated with or without CDDP (20 μM) for 3 h. Then, cells were performed to 50 mM of DCFH-DA (Sigma Chemical, St. Louis, MO, United States) for 40 min at 37°C and then washed with PBS. The intracellular ROS level was measured by a Microplate reader at excitation and emission wavelengths of 488 and 535 nm. The fluorescence was immediately detected using a fluorescence microscope.

### JC-1 Assay

Mitochondrial membrane potential (MMP) was assessed using JC-1 staining. mTECs grown in 12-well plates were incubated with Iso (5, 10, or 20 μM) for 1 h, then treated with CDDP (20 μM) for 18 h and then washed with PBS. Next, cells were incubated with JC-1 (the Beyotime Institute of Biotechnology) for 20 min at 37°C. The fluorescence was detected using flow cytometry.

### Animals

C57BL/6 WT mice were obtained from Liaoning Changsheng Technology Industrial, Co., Ltd. (Certificate SCXK2010-0001; Liaoning, China). Nrf2 knockout mice on a C57BL/6 background were purchased from The Jackson Laboratory (Bar Harbor, ME, United States). All mice were housed in a Specific pathogen free-facility with a 12-h dark/12-h light circle, allowed free access to standard rodent chow and water unless specifically indicated. Animal studies were approved by the Animal Welfare and Research Ethics Committee of Jilin University.

### Experimental Models of AKI

The experimental mice were divided into four groups: control (saline), Iso (50 mg/kg), CDDP only (20 mg/kg; MCE, United States) and CDDP (20 mg/kg) + Iso (50 mg/kg). The mice were fasted, but they provided with tap water *ad libitum* for 12 h prior to Iso (50 mg/kg) i.p. administration for 3 consecutive days. One hour after the first Iso treatment, they were exposed to CDDP (20 mg/kg) for 72 h, then blood and kidney tissues were subsequently collected to evaluate pathological changes and perform biochemical analyses.

### Histopathological Assessment

Mouse fresh kidneys were fixed in Neutral-buffered formalin and paraffin-embedded sections were stained with H&E, pathological changes were analyzed using a microscope. The pathological index in H&E stained kidney sections was scored based on the percentage of tubules with necrosis, detachment, cast formation, dilation, or cell swelling.

### Biochemical Assay

For renal function analysis, BUN and serum creatinine levels were assessed using an assay kit according to the manufacturer’s instructions (Nanjing Jiancheng Bioengineering Institute, Nanjing, China). In addition, the GSH, SOD, MDA, and MPO levels from kidney tissues were quantified according to the manufacturer’s instructions (Nanjing Jiancheng Bioengineering Institute, Nanjing, China).

### Western Blot Analysis

Kidney tissues and mTECs were homogenized in RIPA lysis buffer that contained protease and phosphatase inhibitors. Nuclear and cytoplasmic protein of kidney tissues were obtained using an NE-PER Kit (Pierce Biotechnology, Rockford, IL, United States) according to the manufacturer’s procedure. Protein concentrations were determined by BCA protein assay kit (Beyotime, China). Samples were separated by SDS-poly-acrylamide gel and transferred onto PVDF membranes. The membranes were blocked with 5% non-fat milk. Then the membranes were incubated with primary antibodies overnight at 4°C, subsequently washed and incubated with secondary antibodies. Immunoreactive bands were revealed with ECL. Immunoreactive bands density was performed using ImageJ software.

### Statistical Analysis

All data are represented as means ± SEM. Statistical analysis of the data are implemented by one-way ANOVA. *P* < 0.05 was considered to significant differences.

## Results

### Iso Protects Against CDDP-Induced Cell Death and Apoptosis in mTECs

**FIGURE 1:**
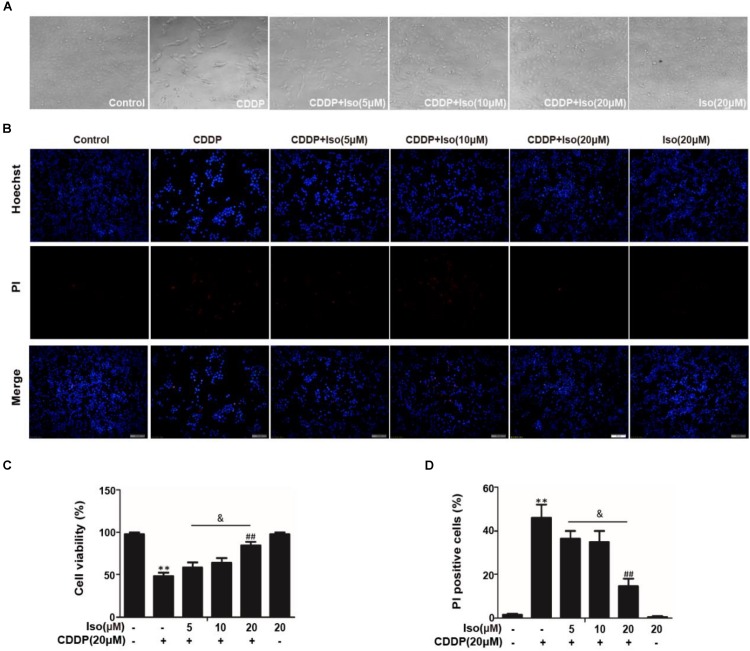
Effects of Iso on CDDP-induced cell viability and apoptosis in mTECs. mTECs were administered various concentrations of Iso (5, 10, and 20 μM) for 1 h, and the cells received CDDP (20 μM) for 24 h. **(A,C)** Cellular morphology and the structure of mTECs was assessed by bright field (40×magnification) microscopy, and cell viability was analyzed by the CCK-8 assay. **(B,D)** Staining with Hoechst/PI was performed for 15 min. The fluorescence was immediately detected by fluorescence microscopy, and the number of PI-positive cells was calculated. The results show the average of three independent experiments. **p < 0.01 versus the control group; ^##^*p* < 0.01 versus the CDDP group; and *p* < 0.05 versus the Iso (20 μM) plus CDDP group.

Our previous studies screened numerous activators of Nrf2, and we found that Iso, a natural product, counteracts oxidative stress by regulating the Nrf2/ARE pathway (Fan et al., 2018). Encouraged by these observations, we utilized this antioxidant molecule and investigated its protective effect on CDDP-induced nephrotoxicity. The protective effect of Iso was first examined in mTECs. The data in Figures 1A,C show that the cell viability of the mTECs treated with CDDP and Iso increased compared to that in the group treated with CDDP only. Moreover, CDDP-induced cytotoxicity was weakened in a dose-dependent manner when the cells were treated with Iso. Iso at a concentration of 20 μM also largely reduced the CDDP-induced increase in the number of PI-positive cells and apoptotic cells (Figures 1B,D). These results demonstrate that Iso has a protective effect on CDDP-induced mTECs death.

### Iso Activates SIRT1, SIRT6, and the Nrf2 Antioxidant Pathway and Reduces CDDP-Induced ROS Generation in mTECs

**FIGURE 2:**
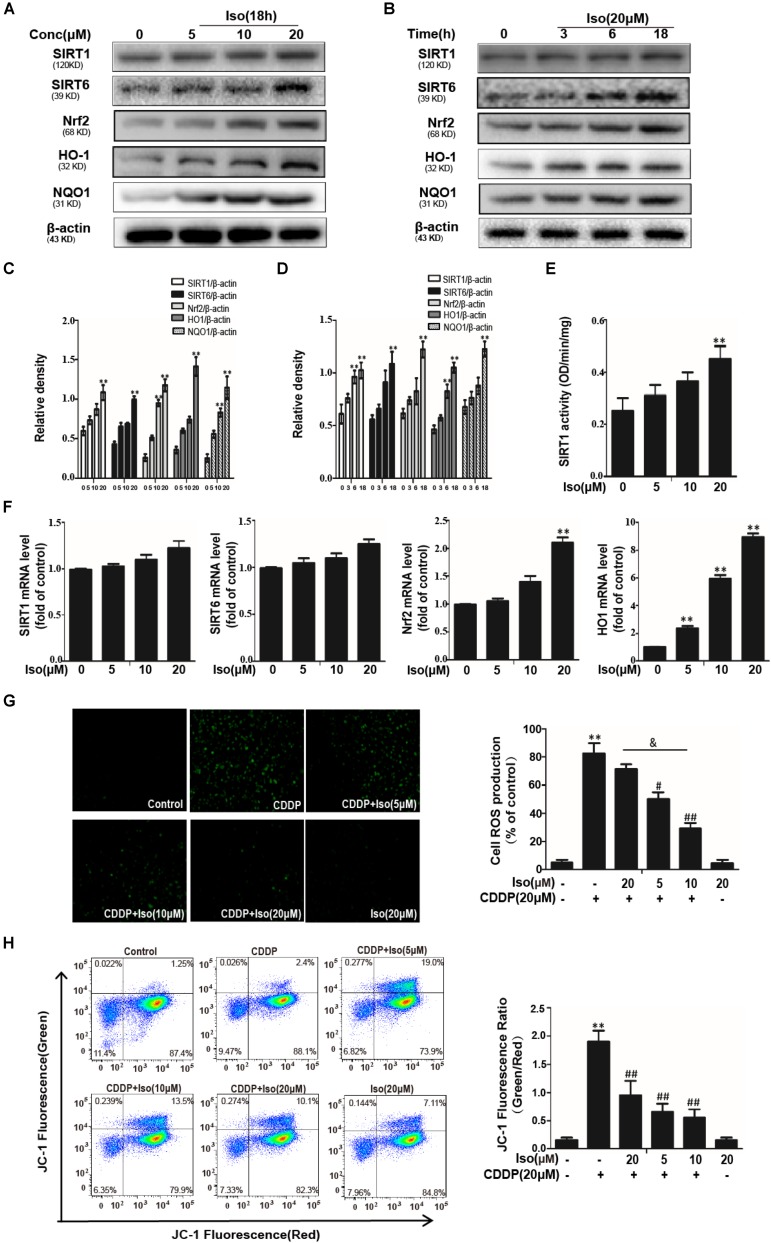
Effects of Iso on CDDP-induced ROS levels and antioxidant pathway in mTECs. **(A–D)** mTECs were treated with Iso at the indicated concentration and for the indicated time, and Western blot analysis was used to determine the protein levels. **(E)** The enzymatic activity of SIRT1 in mTECs. **(F)** Effects of Iso on SIRT1, SIRT6, Nrf2, and HO1 genes expression. Total RNA was extracted from mTECs and genes expression was quantified using real-time PCR. **(G)** mTECs were treated with Iso (5, 10, and 20 μM) for 18 h, treated with CDDP (20 μM) for 10 min, and then stained with 50 μM DCFH-DA for 40 min. The fluorescence was immediately detected by a fluorescence microscope and a multidetection reader. **(H)** The effects of Iso on mitochondrial membrane potential were tested using the JC-1 method and were determined by flow cytometry and a fluorescent microscope. **p < 0.01 versus the control group; ^#^*p* < 0.05 and ^##^*p* < 0.01 versus the CDDP group; and *p* < 0.05 versus the Iso (20 μM) plus CDDP group.

The data in Figures 2A–D show that Iso significantly up-regulated the expression levels of SIRT1, SIRT6, and Nrf2 in a dose- and time-dependent manner as well as increased the expression levels of antioxidant enzyme. And enzymatic activity of SIRT1 was significantly increased compared with in control (Figure 2E). But not all these genes were increased in mTECs (Figure 2F). The potential involvement of ROS in the protective of Iso against CDDP-induced mTECs death was subsequently examined. When Iso was administered, it clearly diminished CDDP-induced intracellular ROS generation (Figure 2G). The data in Figure 2H showed that Iso enhanced the CDDP-induced reduce of MMP in a dose-dependent manner. These results demonstrate that Iso improved CDDP-induced mitochondrial dysfunction in mTECs.

### Iso Suppresses the Activation of Oxidative Stress, Inflammation, and the Apoptosis Pathway in mTECs Treated With CDDP

**FIGURE 3:**
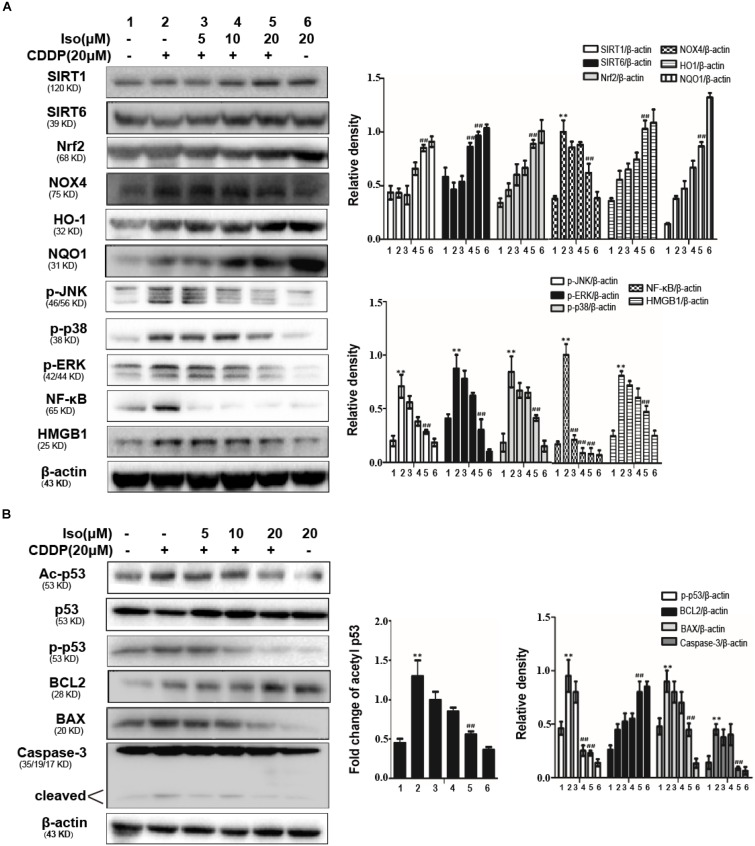
Effects of Iso on CDDP-induced oxidative stress, inflammation and the apoptosis pathway in mTECs. **(A,B)** mTECs were treated with various concentrations of Iso (5, 10, and 20 μM) for 1 h, and the cells were administered CDDP (20 μM) for 24 h. Whole-cell lysates were collected for Western blot to determine the protein levels. Representative Western blots and the statistical results are shown. **p < 0.01 versus the control group; ^##^*p* < 0.01 versus the CDDP group.

Iso up-regulated the expression levels of SIRT1 and SIRT6 *in vitro*. In addition, Iso activated Nrf2 translocation and the expression levels of its regulated antioxidant enzymes, HO-1 and NQO1, whereas it inhibited the expression level of NOX4, thus decreasing oxidative stress and mitochondrial dysfunction. Moreover, Iso attenuated the CDDP-induced activation of HMGB1 and the phosphorylation of JNK, p38, ERK and NF-κB to suppress inflammation and also decreased the CDDP-induced up-regulation of cleaved caspase-3 and the BAX/BCL2 ratio to inhibit apoptosis (Figures 3A,B). It has been shown that acetylation of p53 is a key roles in transcriptional activation of inflammation-related genes and pathological conditions associated with apoptosis. SIRT1 and SIRT6 were localized in the nucleus can deacetylate p53 (Taira and Yoshida, 2012). Therefore, we investigated the acetylation level of p53 following CDDP+ Iso or CDDP only administration to indirectly estimate SIRT1 activity in mTECs. As shown in Figure 3B, compared to the control, CDDP treatment shown a significant increase in the acetylation level of p53. Compared to CDDP treatment alone, Iso treatment markedly reduced the expression of the acetylated forms.

### The Expression of SIRT1 and SIRT6 Protects mTECs From CDDP-Induced Cell Death Through the Activation of NRF2

**FIGURE 4:**
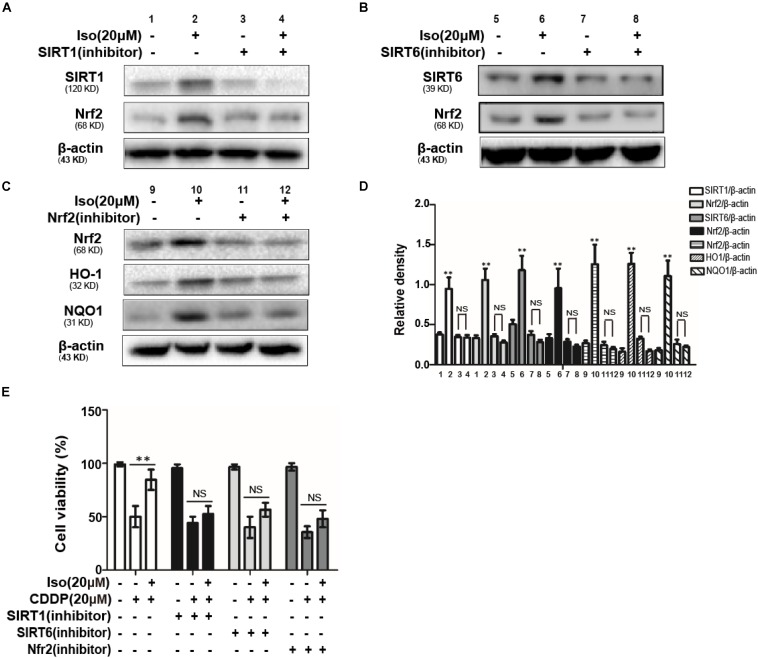
Effects of SIRT1 and SIRT6 on CDDP-induced cytotoxicity and the regulation of Nrf2 in mTECs. **(A,B)** The effects of Iso on the protein levels of SIRT1, SIRT6, and Nrf2 in mTECs after treatment with SIRT1 and SIRT6 inhibitors. **(C)** The effects of Iso on the expression levels of Nrf2, HO1, and NQO1 in mTECs after treatment with a Nrf2 inhibitor. **(D)** SIRT1, SIRT6, Nrf2, HO1, and NQO1 signal intensities were quantified. **(E)** The effects of Iso on the *in vitro* viability of mTECs treated with or without SIRT1, SIRT6, and a Nrf2 inhibitor. **p < 0.01 versus the control group; NS, not significant.

To further examine the relationship between SIRT1 and SIRT6 and the activation of Nrf2, SIRT1, and SIRT6 inhibitors were added. The expression levels of SIRT1 and SIRT6 as well as that of Nrf2 was inhibited after 18 h of coactivation with either of these two inhibitors, as indicated by Western blot in mTECs (Figures 4A–C). In addition, The effects of SIRT1, SIRT6, and Nrf2 inhibitors on CDDP-induced cytotoxicity was studied by the CCK-8 assay, which indicated that the effect of Iso administrate on CDDP-induced cytotoxicity may be related to the expression levels of SIRT1 and SIRT6 and the activation of Nrf2 (Figure 4E).

### Iso Ameliorates CDDP-Induced AKI in Mice

**FIGURE 5:**
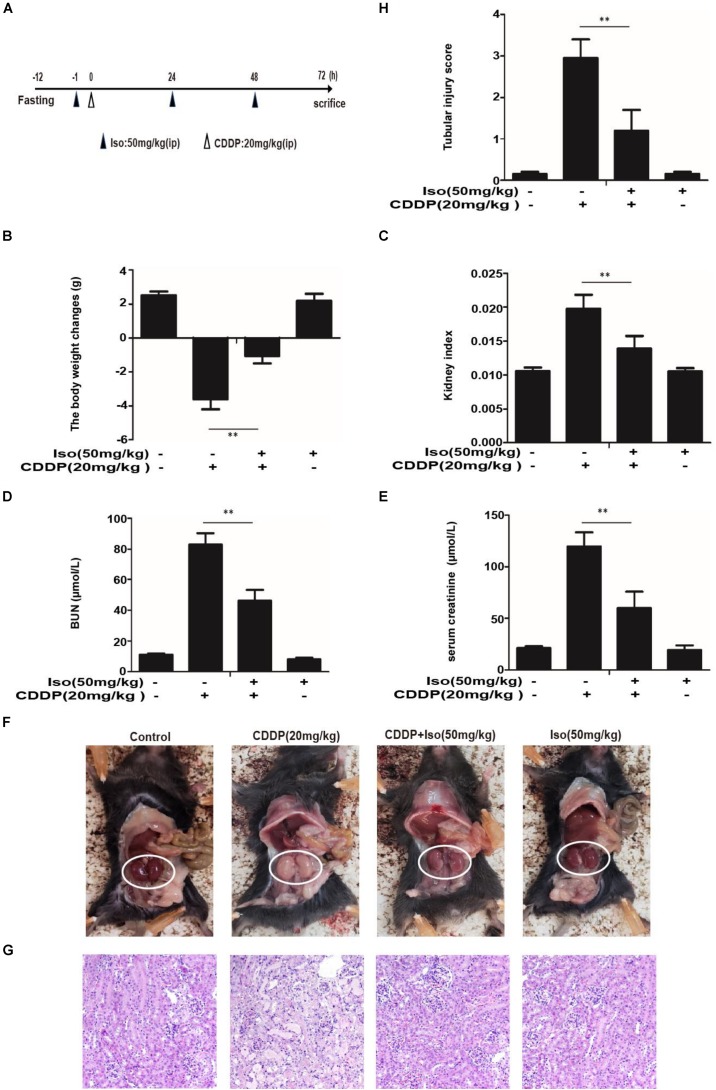
Effect of Iso on CDDP-induced renal dysfunction. **(A)** Experimental study design and time chart for CDDP alone and combined with Iso treatments. After fasting for 12 h, mice were treated with 50 mg/kg Iso and administered 20 mg/kg CDDP after 1 h via ip injection. Iso was subsequently administrated twice at 24 and 48 h post-injection, respectively. Mice were sacrificed at 24 h after the last Iso administration. **(B)** Body weight changes were calculated by subtracting body weights at 72 h with body weights at –1 h. **(C)** Kidney indexes were expressed as kidney weights divided by body weights at 72 h. **(D,E)** BUN and serum creatinine were measured to estimate kidney dysfunction caused by CDDP. **(F)** Representative macroscopic mouse kidney. **(G)** Kidney specimens were stained with H&E. **(H)** Tubular injury was scored using the quantitative evaluation method as described in the section “Materials and Methods” **p < 0.01.

Encouraged by these observations in mTECs, we investigated the protective effect of this antioxidant molecule on CDDP-induced nephrotoxicity in mice. After fasting for 12 h, a single dose of CDDP (20 mg/kg) in mice induced AKI or combined with three ip administrations of Iso (Figure 5A). As indicated in Figures 5B–H, Iso treatment significantly reduced the CDDP-mediated loss of body weight, increasing of the kidney index and the levels of serum BUN and creatinine, swelling, and histopathological injury in kidney tissue.

### Iso Decreases Oxidative Stress in CDDP-Induced AKI in Mice

**FIGURE 6:**
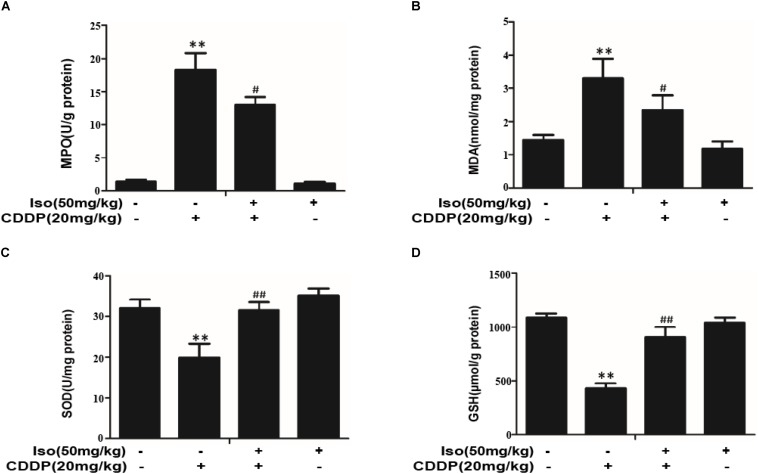
Effects of Iso on oxidative stress in CDDP-induced acute kidney injury. **(A–D)** Effects of Iso on MPO, MDA, SOD, and GSH levels in renal tissues from mice. **p < 0.01 versus the control group; ^#^*p* < 0.05 and ^##^*p* < 0.01 versus the CDDP group.

As oxidative injury plays a vital role in CDDP-induced AKI in mice, the levels of SOD, GSH, MPO, and MDA were investigated whether Iso treatment can prevent nephrotoxicity by suppressing oxidative stress. MPO and MDA levels were lowered by pretreatment with Iso (Figures 6A,B), and SOD and GSH levels were significantly increased (Figures 6C,D).

### Iso Decreases Oxidative Stress, Inflammation, and Apoptosis in CDDP-Induced AKI in Mice

**FIGURE 7:**
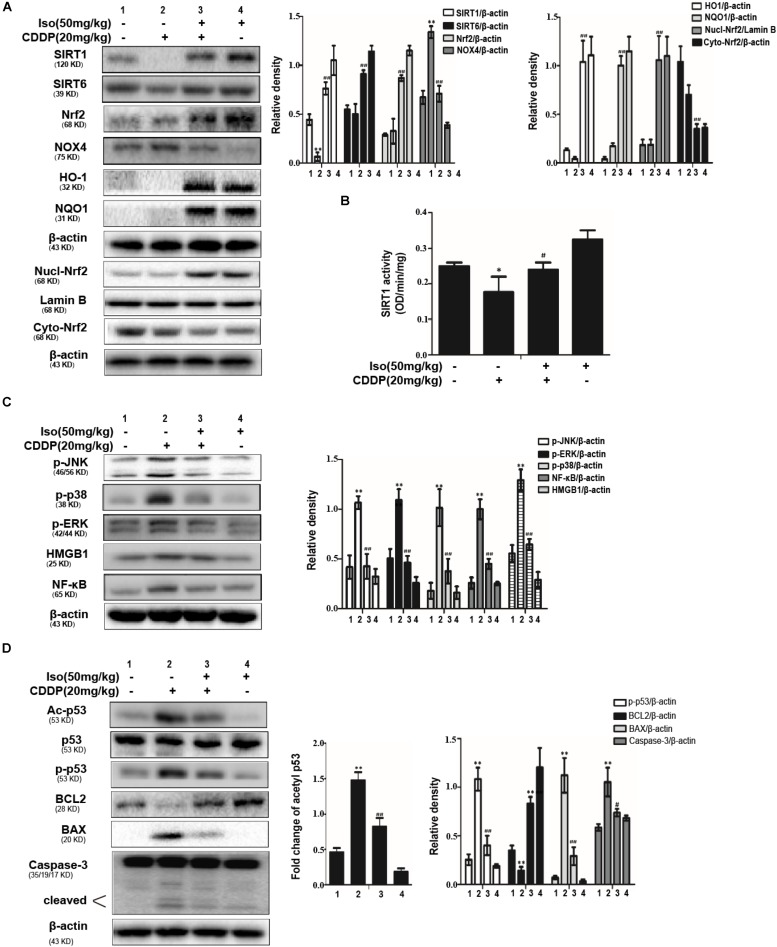
Effects of Iso on the protein expression of oxidative stress, inflammation and apoptosis in CDDP-induced acute kidney injury. **(A)** Representative Western Blots and statistical results show the expression levels of SIRT1, SIRT6, total Nrf2, nuclear Nrf2, cytoplasmic Nrf2, HO-1, NQO1, and NOX4 in renal tissues from mice. **(B)** The enzymatic activity of SIRT1 in the kidney. **(C)** Representative Western Blots and statistical results show the expression levels of phosphorylated JNK, p38, ERK, and HMGB1, NF-κB in renal tissues from mice. **(D)** Representative Western Blots show the expression levels of Ac-p53, p53, p-p53, BCL2, BAX, and cleaved Caspase-3 in the kidneys. *p < 0.05 and **p < 0.01 versus the control group; ^#^*p* < 0.05 and ^##^*p* < 0.01 versus the CDDP group.

To explore the mechanism underlying the protective effect of Iso in AKI, Western blot was used to identify oxidative stress, inflammation and apoptosis in kidney tissue after CDDP injection. The results indicated that Iso treatment markedly enhanced the expression levels of SIRT1, SIRT6, Nrf2, HO-1, and NQO1 compared with that in the CDDP-exposed group. Moreover, CDDP increased the expression level of NOX4 in kidney tissue, while Iso pretreatment suppressed this phenomenon (Figure 7A). Next, it was checked whether the activity of SIRT1 is related to the regulation of Iso. Our results found that Iso increased SIRT1 activity, as indicated by direct enzymatic activity testing (Figure 7B).

It has been reported that the inflammatory pathway plays an important role in CDDP-induced nephrotoxicity, especially the NF-κB and MAPK inflammatory pathways, our experiment determined the effects of Iso treatment on the CDDP-activated NF-κB signaling pathway. As presented in Figure 7C, Iso treatment noticeably reduced the expression levels of NF-κB, HMGB1, JNK, ERK, and p38 phosphorylation compared to that in the CDDP challenged group, which suggests that Iso inhibited inflammatory responses may be partly responsible for blocking the activation of the NF-κB, HMGB1, and MAPK signaling pathways. It has been reported that p53 plays a key role in the expression of apoptosis-related genes during CDDP-related stress (de Haan, 2011; Tsai et al., 2011). The expression levels of cleaved caspase-3, p-p53, and BAX were all decreased in the Iso+CDDP mice, and BCL2 was significantly increased; moreover, the CDDP mice showed an opposite trend (Figure 7D).

These results indicate that the protective action of Iso treatment on CDDP-induced oxidative stress, inflammation and apoptosis may be related to the enhancement of SIRT1 and SIRT6 expression, the up-regulation of Nrf2 and its target genes, and the inhibition of the MAPK, NF-κB, and p53 signaling pathways.

### The Effects of Iso on CDDP-Induced AKI Were Dependent on Nrf2

**FIGURE 8:**
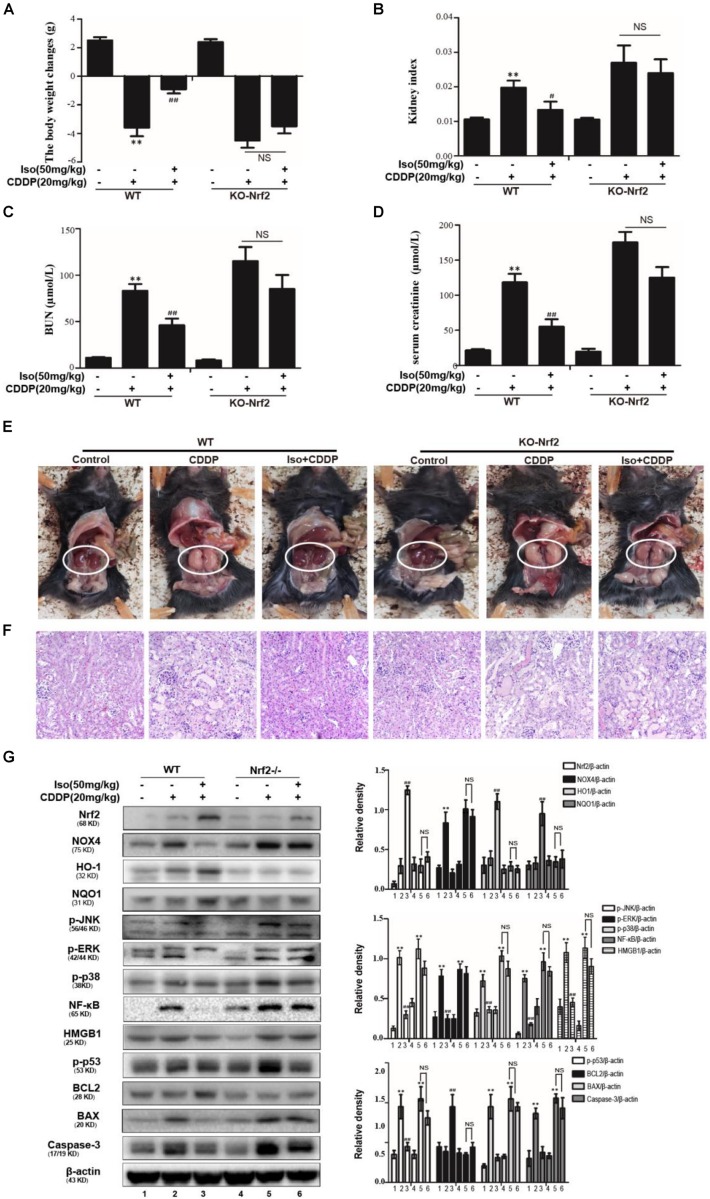
Effects of Iso-mediated regulation of Nrf2 on CDDP-induced acute kidney injury in mice. After fasting for 12 h, WT and Nrf2^–/–^ mice were intraperitoneally injected with 50 mg/kg Iso and 20 mg/kg CDDP after 1 h. Iso was subsequently administrated twice at 24 and 48 h post-injection, respectively. Mice were sacrificed at 24 h after the last Iso administration. **(A)** Body weight changes of WT and Nrf2^–/–^ mice were calculated by subtracting body weights at 72 h with body weights at –1 h. **(B)** Kidney indexes of WT and Nrf2^–/–^ mice were expressed as kidney weights divided by body weights at 72 h. **(C,D)** Creatinine and BUN of WT and Nrf2^–/–^ mice were measured to estimate kidney dysfunction caused by CDDP. **(E)** Representative macroscopic kidney in WT and Nrf2^–/–^ mice. **(F)** Kidney specimens of WT and Nrf2^–/–^ mice were stained with H&E. **(G)** Effects of Iso on the protein expression of WT and Nrf2^–/–^ mice. All data represent the mean ± SEM (*n* = 5 per group). *p < 0.05 and **p < 0.01 versus the CDDP group; ^##^*p* < 0.01 versus the control group; NS, not significant.

We employed WT and Nrf2^–/–^ mice to determine whether the protective action of Iso on CDDP-induced AKI is dependent on Nrf2 activation. Kidney function was evaluated by the body weight, the kidney index, the levels of serum BUN and creatinine, macroscopic kidney and histological examinations. The results showed that Iso treatment effectively attenuated AKI in the WT mice but that this effect was clearly mitigated in the Nrf2^–/–^ mice (Figures 8A–F). We therefore examined whether the antioxidant, anti-inflammatory and anti-apoptotic activities of Iso are associated with the up-regulation of the Nrf2-mediated signaling pathway. Importantly, Iso treatment in WT mice increased the expression levels of Nrf2, HO1, and BCL2 but significantly inhibited the expression of these proteins in Nrf2^–/–^ mice, whereas the phosphorylation of JNK, p38, ERK, NF-κB, and HMGB1 was exacerbated in the kidneys of the knockout mice after CDDP injection (Figure 8G). However, Iso treatment in the Nrf2^–/–^ mice, in contrast to the WT mice, did not alleviate CDDP-induced AKI. These results indicate that Iso plays a vital role in the reduction of oxidative stress, inflammation and apoptosis in CDDP-induced nephrotoxicity, which may be dependent on the upregulation of Nrf2. In conclusion, there is growing evidence demonstrates that Nrf2-mediated signaling pathways are crucial for inhibiting CDDP-induced oxidative stress, inflammation, and apoptosis.

## Discussion

Cisplatin is an effective anticancer drug used in clinical practice, but it has serious complications, especially AKI (Kang et al., 2011; Sahu et al., 2013). In this study, we showed the potential of Iso to prevent CDDP-induced AKI. Natural products have lots of advantages over conventional chemical compound-based drugs, including fewer side effects, less long-term toxicity, variable bioavailability, and biological activity, and more (Kim, 2018), they have significant contributions to drug discovery. Previous studies have been shown that Iso, as a flavonoid-like compound, exerts various pharmacological activities, such as anti-inflammatory activities and antioxidant activities (Anilkumar et al., 2017). Our previous studies screened numerous activators of Nrf2, and we found that Iso ameliorates APAP-induced hepatotoxicity via the activation of the Nrf2 antioxidative pathway and the involvement of AMPK/Akt/GSK3b (Fan et al., 2018). To date, there has been no report on the protective effect of Iso on CDDP-induced nephrotoxicity. Encouraged by these observations, we investigated the protective effect of this antioxidant molecule on CDDP-induced nephrotoxicity. In this study, we demonstrated that Iso protected against CDDP-induced renal damage in mice and cell damage in mTECs, as shown by body weight, the kidney index, serum levels of BUN and creatinine, histopathological changes, cell viability, ROS, and apoptosis. These data suggest that Iso can attenuate CDDP-induced acute nephrotoxicity. We further explored the underlying molecular mechanisms *in vitro* and *in vivo*. Our results support the possibility that Iso attenuates CDDP-induced nephrotoxicity via the SIRT1/SIRT6-Nrf2 pathway.

mTECs injury leads to subsequent nephrotoxicity upon CDDP treatment, and the protective effect of Iso was first studied in mTECs. The cell protection mediated by Iso is dose-dependent. In addition, Iso markedly reduced the CDDP-induced ROS levels in mTECs (Figure 2G) as well as in kidney tissue. The generation of MPO and MDA and the depletion of SOD and GSH have frequently been used as indicators of oxidative stress. In our study, high levels of MPO and MDA and low levels of SOD and GSH are characteristics of oxidative stress induced by CDDP, and these alterations were reversed by Iso (Figures 6A–D). Thus, Iso may protect against CDDP-induced nephrotoxicity by suppressing oxidative stress. Moreover, CDDP-induces ROS to activate multiple signaling pathways, such as MAPKs, NF-κB, and p53 mediated pathways, thus exacerbating its side effects (Sung et al., 2008). We then investigated the possible mechanisms underlying the protective effect of Iso. It is well known that Nrf2 is the main defense mechanism against cellular oxidative stress. There have been reports of studies related to the role of Nrf2, especially the up-regulation of Nrf2 to prevent CDDP-induced nephrotoxicity as a strategy (Park et al., 2008; Aleksunes et al., 2010). In addition, Nrf2 expression and its nuclear translocation were also positively correlated with the induction of the antioxidant defense and phase II detoxifying enzymes (Xu et al., 2014). Our results suggest that Iso up-regulated the expression levels of Nrf2, HO-1, and NQO1 and downregulated the expression level of the ROS-generating protein NOX4 in mTECs and in mice (Figures 3A, 7A). Many studies have shown that SIRT1 and SIRT6 exert potent antioxidant effects by enhancing the transcriptional activity of Nrf2 (Zhang et al., 2017). In this study, Iso significantly increased the expression levels of SIRT1 and SIRT6 in mTECs and in mice, which may have contributed to activation of Nrf2. Our findings confirm the hypothesis that inhibitors of SIRT1 and SIRT6 abolished the activation of Nrf2 and the cytoprotection of Iso in mTECs (Figure 4). Taken together, these results suggest that the nephroprotective effect of Iso may be attributable to its antioxidant properties through increasing the expression levels of SIRT1 and SIRT6 and further activating the Nrf2 pathway.

It has been shown that acetylation of p53 is a key roles in transcriptional activation of inflammation-related genes and pathological conditions associated with apoptosis. SIRT1 and SIRT6 were localized in the nucleus can deacetylate p53 (Taira and Yoshida, 2012). Therefore, we investigated the acetylation level of p53 following CDDP + Iso or CDDP only administration to indirectly estimate SIRT1 activity in mTECs and in mice. We found CDDP treatment significantly increased p53 acetylation level. And Iso treatment markedly blocked the expression of the acetylated forms (Figures 3B, 7D). Of course, the enzymatic activity of SIRT1 was significantly increased by Iso in mTECs and in mice (Figures 2E, 7B).

Previous research has reported that apoptosis, necrosis and necroptosis were induced by CDDP in proximal tubular cells, especially apoptosis, which is a common feature of CDDP-induced nephrotoxicity (Faubel et al., 2004). Besides, CDDP can activate p53, BAX, and caspase-3, leading to the apoptosis of renal tubular cells, while BCL2 can inhibit BAX activity and thereby reduce mitochondrial damage and CDDP-induced apoptosis (Lee et al., 2001; Cummings and Schnellmann, 2002; Jiang et al., 2004; Wei et al., 2007). The pro-apoptotic BAX protein can be directly activated by p53was mentioned in some research (Chipuk et al., 2004). In this research, CDDP activated p53, BAX, and caspase-3 and reduced BCL2 expression in mTECs and in mice (Figures 3B, 7D). This phenomenon was reversed by Iso treatment (Figures 3B, 7D). These results indicate that Iso exhibits anti-apoptotic by inhibiting the expression levels of p53, BAX, and caspase-3 and increasing the expression level of BCL2.

The serine/threonine kinases cascades that regulate cell survival and death is MAPK pathway (Chang and Karin, 2001). Moreover, CDDP has been shown to activate ERK, JNK and p38 *in vitro* and *in vivo*, and inhibit MAPK activity can inhibit tubular apoptosis and CDDP-induced renal toxicity (Ozkok and Edelstein, 2014). In this study, Iso significantly reduced the phosphorylation of JNK, ERK, and p38 *in vitro* and *in vivo* (Figures 3A, 7C). The mechanism by which NAD+/SIRT1 protects the kidneys involves the epigenetic regulation of the JNK pathway (Guan et al., 2017). In addition, SIRT6 regulated inflammation and apoptosis by inhibiting ERK1/2 signaling in CDDP-induced AKI (Li et al., 2018). On the basis of previous reports, our results imply that Iso inhibited CDDP-induced inflammation may be associated with SIRT1-SIRT6/Nrf2 activation.

To identify the target of Iso in CDDP-induced nephrotoxicity, we initially focused on Nrf2. We hypothesized that Iso can induce Nrf2 activation and translocation from the cytoplasm to the nucleus via SIRT1 or SIRT6 activation and consequently inhibit inflammation, oxidative stress and apoptosis. The data in Figure 4 shown that Iso-induced Nrf2 activation and ameliorated of CDDP-induced cytotoxicity were abolished by a SIRT1 or SIRT6 inhibitor, which suggests that Iso exerts protective effects against CDDP-induced toxicity *in vitro* through Nrf2 activation via the SIRT1/SIRT6 pathway. This study used Nrf2^–/–^ mice to further elucidate whether the antioxidant activity of Iso is directly related to Nrf2 activation in CDDP-induced AKI and the existence of a potential relationship. The data in Figure 8 indicate that Iso protects mice from CDDP-induced renal damage in WT mice, as evidenced by its effects on weight loss, the kidney index, serum levels of BUN and creatinine, and histopathological changes; however, this phenomenon effectively abolished in Nrf2^–/–^ mice. Moreover, the Iso-mediated increases in the expression levels of Nrf2, HO-1, and NQO1 and the subsequent inhibition of the inflammatory and apoptotic signaling pathways in WT mice were significantly abolished in Nrf2^–/–^ mice (Figure 8). Together, our results support the possibility that Iso attenuated CDDP-induced AKI via the SIRT1/SIRT6-Nrf2 pathway.

In conclusion, the current study provides a comprehensive understanding of the therapeutic potential of Iso in CDDP-induced nephrotoxicity and the possible mechanisms involved. Our results indicate that the absence of Nrf2 exacerbates CDDP-induced renal damage and that the pharmacological activation of Nrf2 may represent a novel therapy for preventing kidney injury. In this study, Iso was shown to be effective in ameliorating CDDP-induced AKI via the SIRT1/SIRT6-Nrf2 pathway, indicating that Iso may be a novel and promising therapeutic agent.

## Data Availability Statement

All datasets generated for this study are included in the article/supplementary material.

## Ethics Statement

All animal studies were reviewed and approved by the Animal Welfare and Research Ethics Committee of Jilin University.

## Author Contributions

XF conducted the experiments and wrote the manuscript. WW, JH, and XL conducted the experiments. XC contributed to the design and improvement of the experiments.

## Conflict of Interest

The authors declare that the research was conducted in the absence of any commercial or financial relationships that could be construed as a potential conflict of interest.
